# Adsorption and Desorption Properties of Total Flavonoids from Oil Palm (*Elaeis guineensis* Jacq.) Mature Leaf on Macroporous Adsorption Resins

**DOI:** 10.3390/molecules25040778

**Published:** 2020-02-11

**Authors:** Mohamad Shazeli Che Zain, Soo Yee Lee, Chian Ying Teo, Khozirah Shaari

**Affiliations:** 1 Laboratory of Natural Products, Institute of Bioscience, Universiti Putra Malaysia, 43400 Serdang, Selangor, Malaysia; shazelizain@gmail.com (M.S.C.Z.); daphne.leesooyee@gmail.com (S.Y.L.); 2 Department of Pharmaceutical Chemistry, School of Pharmacy, International Medical University, 57000 Kuala Lumpur, Malaysia

**Keywords:** oil palm (*Elaeis guineensis* Jacq.) leaf, total flavonoids, macroporous resins, adsorption, desorption, antioxidant activity

## Abstract

Three different macroporous resins (XAD7HP, DAX-8, and XAD4) were evaluated for their adsorption and desorption properties in preparing flavonoid-enriched oil palm (*Elaeis guineensis* Jacq.) leaf extract. The influences of initial concentration, solution pH, contact time, and desorption solvent (ethanol) concentration were determined by static sorption/desorption methods. The optimal condition for adsorption of flavonoids was achieved when the solution of the extract was adjusted to pH 7, reaching equilibrium after 1440 min at 298 K. The adsorption process was well described by a pseudo-second-order kinetics model, while the adsorption isotherm data fitted well with a Freundlich model. The adsorption by each resin was via an exothermic and physical adsorption process. Based on the static experiment results, XAD7HP was found to be the most appropriate adsorbent, while 80% ethanol was the best solvent for desorbent. Further evaluation of its dynamic adsorption and desorption characteristics on a packed glass column showed that XAD7HP could enrich the OPL total flavonoid content by a 3.57-fold increment. Moreover, UHPLC–UV/PDA and UHPLC–MS/MS analysis revealed that apigenin and luteolin derivatives were selectively adsorbed by XAD7HP. Additionally, both the crude OPL extract and the flavonoid-enriched fraction have good DPPH and NO free radical scavenging activities. Multiple interactions between the flavonoids and cross-linked polymeric XAD7HP resin through van der Waals forces and hydrogen bonding described the sorption processes. Therefore, by utilizing this method, the flavonoid-enriched fraction from crude OPL extract could be used as a potential bioactive ingredient in nutraceutical and pharmaceutical applications at minimum cost with optimum efficiency.

## 1. Introduction

The genus *Elaeis* belongs to the palm family (Arecaceae) which is one of the key members of the allogamous arborescent monocot group under the order Arecales [1]. *Elaeis guineensis* Jacq. is believed to have originated in West Africa [2]. Presently, it is a major commodity crop of some countries in Southeast Asia, notably Indonesia and Malaysia [3]. Oil palm cultivation produces a huge amount of waste material in the form of oil palm trunks, fronds, and leaves. These waste materials, especially oil palm leaves (OPL), contain phytochemicals that could be useful for various applications such as medicines for treating wounds, cancer, and cardiovascular and kidney diseases [4]. OPL extract was shown to be safe for use in disease treatments based on the absence of adverse effects in several studies on rodents [5,6].

Previous chemical investigations revealed that OPL extract is rich in flavonoids, mainly flavonoid *O*-glycosides and flavonoid *C*-glycosides [7]. As a class of compounds, flavonoids are well known to have positive effects on human health with reports of being hepatoprotective, antioxidative, and antidiabetic, among others [8]. Presently, there is a high demand for flavonoids globally by the nutraceutical and pharmaceutical industries, which is forecasted to reach approximately RM 4.40 billion by the year 2025 [9]. Thus, to meet requirements, the availability of simple and efficient methods for their separation and purification is needed.

Conventionally, flavonoids are separated from plant matrixes by methods that require high amounts of solvents, long separation time and low recovery. These methods include solid–liquid extraction, liquid–liquid extraction, and column chromatography, either packed with silica gel or Sephadex LH-20. Macroporous resin (MAR), with the property of high cross-linkage and many permanent pores, was also utilized in the separation and purification of flavonoids, for example, separation of flavonoids from *Lycium barbarum* L. [10], *Populus tomentosa* Carr. [11], *Oleo europaea* L. [12], *Glycyrrhiza glabra* L. [13], *Eriobotrya japonica* (Thunb1.) Lindl. (Loquat) [14], and *Hippophae rhamnoides* L. (Sea buckthorn) [15], among others. The superiority of MARs in separating flavonoids from plant extracts is said to be due to the special characteristics of flavonoids themselves as they have benzene rings and several hydrogen groups attached to their aglycone, helping the appropriate MAR, with proper average pore diameter, polarity, and surface area, to adsorb these compounds. Previous researches showed that such properties significantly contribute to the sorption capacity of MARs [13,16,17]. Therefore, MARs are a suitable medium in devising a simple and effective method for the separation and purification of flavonoids from plant extracts. In the present study, three MARs with different properties (XAD4, XAD7HP, and DAX-8) were evaluated for their adsorption and desorption characteristics, thermodynamics, and kinetics, in the static sorption of total flavonoids from OPL extract. The resin with the best static sorption characteristics was further assessed for purification efficiency based on its dynamic adsorption and desorption characteristics on a packed glass column.

## 2. Results and Discussion

### 2.1. Adsorption Kinetics of OPL Total Flavonoids on Selected MARs

**Figure 1:**
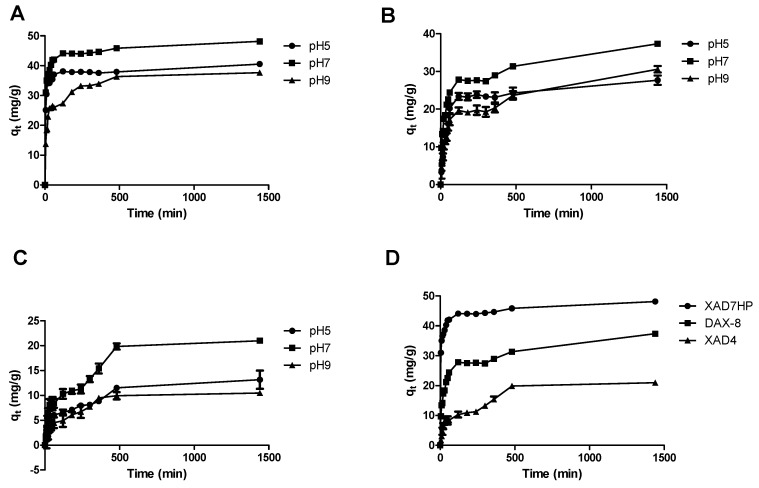
Time curves for adsorption kinetics of the selected MARs for OPL total flavonoids. (**A**) XAD7HP, (**B**) DAX-8, and (**C**) XAD4 at different pH; (**D**) comparison of the time curves for the different MARs at pH 7.

The adsorption kinetics of the MARs for total flavonoids in the OPL extract was firstly investigated in order to determine the equilibrium contact time and optimal pH solution. The solution initial pH value affects the affinity of the flavonoids to the MAR [11]. Hence, in the adsorption kinetics experiment, three pH values (5, 7, and 9) were evaluated. The adsorption curves obtained from the experiments are shown in Figure 1. For the three selected MARs, the adsorption capacity, *q_t_* (mg/g dry MAR), increased with increasing contact time t, reaching equilibrium within 1440 min. For all the resins, pH 7 showed the highest *q_t_* values in comparison to pH 5 and pH 9. This indicated that the adsorption of the OPL total flavonoids on the selected resins was more favorable in neutral conditions than in acidic and basic conditions. This could be due to hydrogen-bonding interactions being reduced at higher pH value. Hydroxyl groups attached to the aglycone were hydrolyzed into hydrogen ions, resulting in low adsorption capacity [18]. Based on the result, the OPL extract solution was adjusted to pH 7 in subsequent experiments.

The pseudo-first-order, pseudo-second-order, and particle diffusion kinetics models were analyzed to elucidate the adsorption behaviors and mechanism of the selected MARs. The derived parameters such as correlation coefficient and dynamic parameters are summarized in Table 1. The pseudo-second-order kinetic model was the more favorable model in demonstrating the adsorption processes of OPL total flavonoids on the selected MARs with good correlation (*R*^2^) values in comparison to the pseudo-first-order kinetics model. Similar results were reported by other authors who also investigated the adsorption of total flavonoids present in various plants on different MARs [10,14,16].

**Figure 2:**
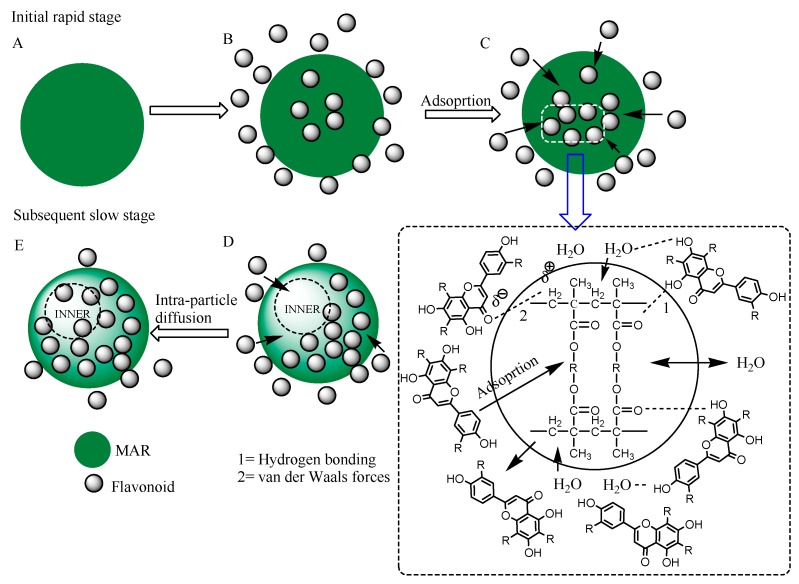
Schematic diagram of the adsorption process of OPL total flavonoids on XAD7HP resin.

The *C*, *k_p_*, and *R*^2^ obtained from the intra-particle diffusion curves of the selected MARs are shown in Table 1. The adsorption of flavonoids on XAD4 was governed by the intra-particle diffusion process, as indicated by the good linear trend over time. However, the diffusion curves for XAD7HP and DAX-8 exhibited weak linear trends over time, which indicated that the adsorption of the flavonoids on XAD7HP and DAX-8 may involve multiple processes. There were three stages involved in the diffusion, comprising boundary layer diffusion (0–10 or 60 min), gradual adsorption (10–120 or 60–1440 min), and equilibrium (120–1440 min) stages. The first stage was attributed to the immediate utilization of the most readily available adsorbing sites on the resin surfaces, while the second stage involved a gradual diffusion of adsorbate from the surface site into the inner pores. The XAD7HP resin underwent a third stage where the diffusion of flavonoids was at equilibrium. The results revealed that the initial portion of flavonoid adsorption by resins may be governed by the initial intra-particle transport of flavonoids controlled by a surface diffusion process, while the later stage is controlled by pore diffusion. For better visualization, a schematic diagram of the whole adsorption process of OPL total flavonoids on XAD7HP resin is illustrated in Figure 2.

Interestingly, for all three resins, the curves did not pass through the origin, which indicated that both boundary layer diffusion and intraparticle diffusion were the rate-controlling factors of the adsorption [14,19]. The particle diffusion kinetics models could not represent the entire adsorption process well because of the weak correlation coefficients [19]. However, it could define a definite mechanism of adsorption at a certain stage.

### 2.2. Adsorption Thermodynamics of OPL Total Flavonoids on the Selected MARs

**Figure 3:**
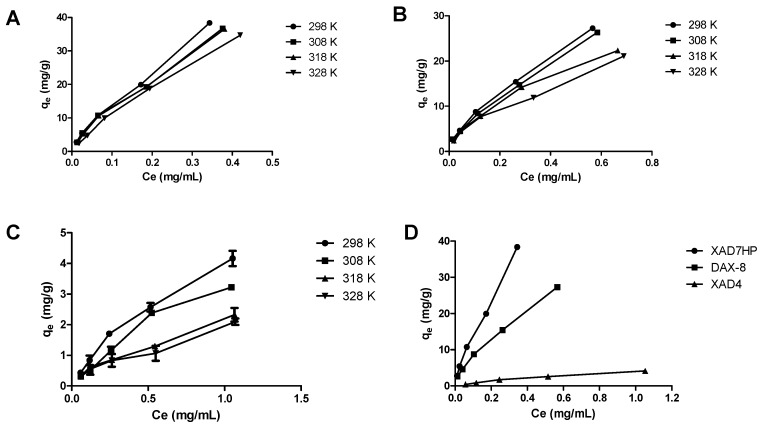
Adsorption isotherms curves of OPL total flavonoids on (**A**) XAD7HP, (**B**) DAX-8, and (**C**) XAD4 at different temperatures; (**D**) comparison of adsorption isotherms of the selected MARs at 298 K.

The equilibrium adsorption isotherms were investigated at 298 K, 308 K, 318 K, and 328 K for each of the selected resins. As shown in Figure 3, it was noticeable that lower temperatures were advantageous for the adsorption of flavonoids on the resins. The curves displayed an upward trend with increasing equilibrium concentration of flavonoids for each of the tested temperatures. However, the slope of the *q_e_* vs. *C_e_* plots declined with an increase in temperature, for all the selected resins. A subsequent investigation was carried out at 298 K since the optimal adsorption capacity was found to be at this temperature.

The adsorption behavior was further assessed using the Langmuir and Freundlich adsorption isotherm models. After model fitting, the Langmuir model gave mostly weak correlation coefficients (*R*_1_^2^) and, therefore, was not suitable to explain the adsorption behavior of XAD7HP, DAX-8, and XAD4 at the different temperatures, except for XAD4 at 298 K, XAD7HP at 328 K, and DAX-8 at 318 K [20]. The Langmuir isotherm model was applied well to many monolayer solid–liquid adsorption processes [21]. The Langmuir constants, *q_m_* and *K_L_*, were calculated from the linear plot *C_e_*/*q_e_* vs. *C_e_* and listed in Table 2. The equilibrium parameter *R_L_*, a dimensionless constant, is a useful parameter to denote the important characteristics of the Langmuir isotherm [22]. The *R_L_* is calculated by the following equation:

(1) $$R_{L}=\frac{1}{1+K_{L}C_{o}},$$

where *C_o_* is the highest initial concentration of flavonoid (mg/mL), and *K_L_* is the calculated value which indicates the affinity between the absorbent and adsorbate molecules. The *R_L_* value gives a sign of the isotherm shape which is either unfavorable (*R_L_* > 1), linear (*R_L_* = 1), favorable (0 < *R_L_* < 1), or irreversible (*R_L_* = 0) [16]. As shown in Table 2, the values of *R_L_* were less than one, so the adsorption of flavonoids onto the MARs was a favorable process.

The Freundlich constants, *K_f_* and 1/*n*, were calculated from the *q_e_* vs. *C_e_* plot. As shown in Table 2, the Freundlich model exhibited good correlation coefficients at different temperatures, which suggested it to be a good model to reflect the adsorption equilibrium of the OPL total flavonoids on the selected MARs. To determine the adsorption intensity or surface heterogeneity, the 1/*n* value was measured [15]. When the value of 1/*n* is greater than one, adsorption is said to be difficult to occur [23]. In the present work, the 1/*n* values were all less than one, which indicated that the adsorption of flavonoids on the selected MARs was favorable. This was in agreement with the results shown by *R_L_*. Based on the Van’t Hoff equation, the enthalpy changes (ΔH) for XAD4, XAD7HP, and DAX-8 were calculated as −19.96 kJ/mol, −7.49 kJ/mol, and −13.41 kJ/mol, respectively. The negative values of enthalpy changes (ΔH) for those resins recommended that the adsorption process was exothermic, and low temperature was good for the adsorption process [24]. Meanwhile, the absolute values of enthalpy changes (ΔH), for the selected MARs, were less than 20 kJ/mol, demonstrating that the adsorption of the OPL total flavonoids on the resin surface was governed by physisorption rather than chemisorption [25].

### 2.3. Static Desorption of OPL Total Flavonoids from the Selected MARs

**Figure 4:**
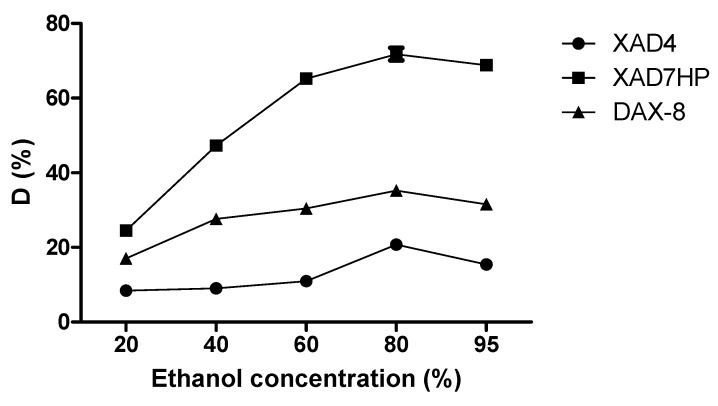
Effect of ethanol concentration on desorption ratio (*D*) of OPL total flavonoids from the selected MARs.

Ethanol is an organic solvent known for several favorable properties, such as it being easily removed from a solution, recyclable, inexpensive, with low toxicity and, therefore, safe for human use [13,26]. Thus, it was selected as a desorbent in the present study. As shown in Figure 4, the desorption ratio of flavonoids was directly proportional to the ethanol concentration, meaning that when the desorption ratio increased, the ethanol concentration also increased. After reaching the peak value, the desorption ratio decreased with a further increase in ethanol concentration. The competing interactions between the intermolecular forces of adsorption on the resins and dissolution in the solvent play a significant role in the desorption of flavonoids from the resins. The flavonoids would be desorbed from the resin into the solvent when intermolecular forces are recessive [11]. It was observed that, at 80% aqueous ethanol, the desorption value was the highest. Hence, a high ethanol concentration (80%) was selected in the dynamic desorption study to ensure complete elution. In comparison with DAX-8 and XAD4, XAD7HP showed the best desorption ratio of about 80% at 80% ethanol concentration. Thus, XAD7HP was selected as the most suitable resin in the dynamic experiments.

### 2.4. Dynamic Adsorption and Desorption of OPL Total Flavonoids from XAD7HP

**Figure 5:**
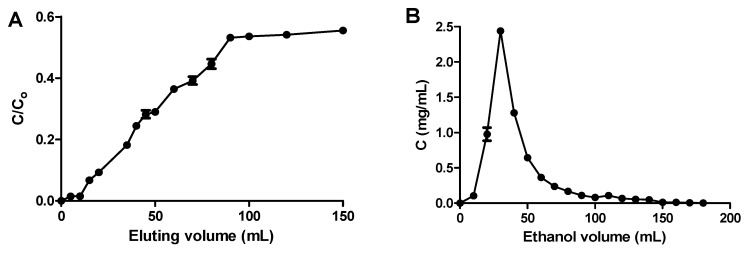
Dynamic breakthrough (**A**) and desorption (**B**) curves for OPL total flavonoids on XAD7HP.

The dynamic breakthrough curve for XAD7HP was constructed based on the eluting volume and the ratio of the TFC of the eluting volume to the TFC of the original OPL extract (*C*/*C_o_*). The breakthrough and the saturation points are represented by 5% and 95% of the *C*/*C_o_* ratio, respectively. As shown in Figure 5A, the breakthrough and saturation points for OPL total flavonoids on XAD7HP were before 15 mL and after 80 mL, respectively. It was also observed that, after the breakthrough point, the adsorption increased rapidly up to the saturation point.

The dynamic desorption curve for XAD7HP was plotted from the volume of the eluting desorption solution and the TFC of the desorbed solution. As displayed in Figure 5B, the OPL total flavonoids could be completely desorbed after elution with 180 mL of 80% aqueous ethanol. The eluted desorbed solution was pooled and evaluated for its TFC. The TFC value of the desorbed solution was 487.97 mg QCE/g, which was a 3.57-fold increase compared to the initial TFC of the original OPL extract of 136.69 mg QCE/g.

### 2.5. UHPLC–UV/PDA and UHPLC–MS/MS Analysis of Crude and Total Flavonoid-Enriched OPL Extracts

**Figure 6:**
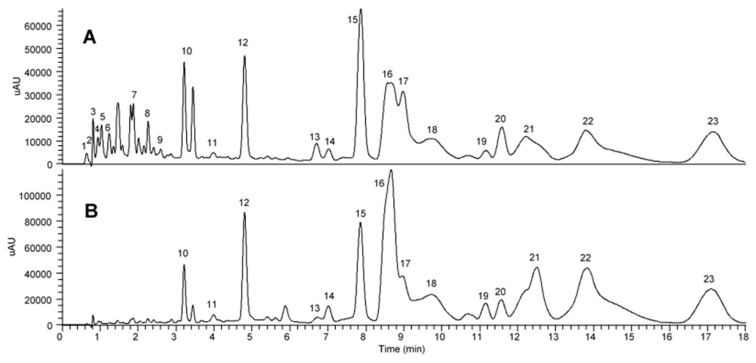
UHPLC–UV/PDA chromatograms of (**A**) crude OPL extract and (**B**) total flavonoid-enriched OPL extract at 340 nm.

Figure 6 reveals the comparison of compounds present in a crude extract and an extract after enrichment with XAD7HP macroporous resin. The peaks were putatively identified after analyzing the fragmentation pattern and UV–Vis absorption spectrum. The assigned peaks were cross-checked with previous literature reports that managed to extensively characterize the metabolites present in crude extract of oil palm leaves [7,27]. Table 3 lists 23 identified compounds ranging from sugar to organic acids, phenolic acids, and flavonoids that were found in crude OPL extract, while 14 of these compounds were found in enriched fraction at different concentration. The crude OPL extract contained sucrose, chelidonic acid dimer, citric acid, and several phenolic acids including dihydroxylbenzoyl-*O*-hexoside, hydroxylbenzoyl-*O*-hexoside, galloyl-*O*-hexoside, vanilloyl-*O*-hexoside, and sinapoyl-*O*-hexoside. These compounds were also reported by Tahir et al. [27]. The rest of the compounds present in crude OPL extract were flavonoids, specifically apigenin and luteolin derivatives [7].

Interestingly, XAD7HP managed to enrich flavonoids present in oil palm leaves and selectively adsorbed apigenin and luteolin derivatives. This was obviously observed in Figure 6 and tabulated in Table 3, whereby the peaks labeled 1–9 were absent and some of the peak intensities of apigenin and luteolin derivatives were increased in comparison with crude OPL extract. including apigenin-6,8-di-*C*-hexose, apigenin-6-*C*-pentose-8-*C*-hexose, isoorientin, apigenin-6-*C*-hexose-8-*C*-pentose, luteolin-6-*C*-hexose-8-*C*-deoxyhexose, vitexin, isovitexin. and apigenin-6-*C*-hexose-8-*C*-deoxyhexose.

### 2.6. Free Radical Scavenging Activities of Crude and Enriched Fraction of OPL

The crude OPL extract and enriched total flavonoid fraction were tested in terms of their antioxidant activities by investigating their capability to inhibit free radicals, which is the main mechanism involved in antioxidant activity. The result is presented as IC_50_ (the concentration required to scavenge 50% of radical) of DPPH and NO free radical scavenging activities in Table 4. Both the crude OPL extract and the flavonoid-enriched fraction possessed good DPPH and NO free radical scavenging activities. However, one unanticipated finding was that the crude OPL which contained total flavonoid of 136.69 mg QCE/g dried extract had better antioxidant capacities than the enriched total flavonoid fraction with 487.97 mg QCE/g dried extract, showing a lower IC_50_ of 15.88 µg/mL in comparison with the IC_50_ of enriched fraction, 59.48 µg/mL. The results were consistent with NO free radical scavenging activity, whereby the crude OPL extract had a higher antioxidant activity with an IC_50_ value of 17.84 µg/mL, while the enriched fraction reached IC_50_ at 68.13 µg/mL. The appropriate explanation for this is due to the synergistic or additive effects. The crude OPL extract contains a wide range of phytochemicals ranging from groups of sugar moieties to phenolic acids, flavonoid glycosides, catechin stereoisomers, fatty acids, organic acids, amino acids, etc. [7,28,29]. Some of these common compounds such as catechin, citric acid, isoorientin, orientin, etc. are well known as having high antioxidative effects; hence, the interaction between these antioxidant constituents can create synergistic or additive effects on scavenging free radicals [27,30], resulting in higher antioxidant activity.

Meanwhile, the macroporous resin was created to selectively adsorb on the compounds present in crude OPL extracts; hence, this study showed that XAD7HP resins tend to adsorb on compounds that have similar characteristics, i.e., flavonoids. There are different groups of flavonoids present in OPL extracts which can be classified into flavone, flavonol, isoflavone, flavanone, etc. [7,29,31]. Although some of these flavonoids such as epicatechin, quercetin, rutin, luteolin, etc. were found to contain a high percentage of free radical inhibition, some of the flavonoids were reported to be poor free radical scavengers such as vitexin and isovitexin [32]. Hence, the combination of these flavonoids could not generate a significant effect on DPPH free radical scavenging activity as compared to the crude OPL extracts. Therefore, the findings could not conclude that the flavonoid-enriched fraction had lower antioxidant capacity than crude OPL extracts solely based on DPPH and NO free radical scavenging activities. Different antioxidants follow different mechanisms or pathways under various stressed conditions in order to show their antioxidative response [27]. Further research should be done to investigate the other mechanisms or pathways of these extracts in expressing their antioxidative response, such as other radical chain reaction inhibitors (FRAP, ABTS, ORAC), metal chelators, quenching single oxygen species, protein/enzymes, antioxidant enzyme cofactors, etc. [27,33,34,35].

### 2.7. Adsorption Mechanism

The adsorption and desorption capacity of total flavonoids from MARs is related to several factors including surface area, average pore size, particle diameter, and polarity [35]. The surface area of XAD7HP (380 m^2^/g) is between that of XAD4 (750 m^2^/g) and DAX-8 (140 m^2^/g). The particle diameters of XAD7HP and XAD4 are the same (0.250–0.841 mm), while DAX-8 is lower than both with a particle diameter of 0.250–0.420 mm. The average pore size of XAD7HP is the largest (300–400 Å) among the tested MARs, followed by DAX-8 (225 Å) and XAD4 (100 Å). The matrix of XAD4 is made of styrene–divinylbenzene, while XAD7HP and DAX-8 are made of acrylic and acrylic ester, respectively. Based on the results obtained, XAD7HP showed the highest adsorption and desorption capacities followed by DAX-8 and XAD4, indicating that large average pore diameter (300–400 Å), medium surface area (380 m^2^/g), and acrylic matrix are the most appropriate properties of MARs for entrapment and release of total flavonoids in OPL extracts.

In addition, the polarity of MARs plays the most vital role in the sorption process. Both XAD7HP and DAX-8 are moderate polar MARs, while XAD4 is a non-polar resin. The results indicated that moderate polar resins (XAD7HP and DAX-8) are most suitable to adsorb and desorb total flavonoids from OPL in comparison to the non-polar resin (XAD4). The polarity matching between OPL extract and moderate polar XAD7HP is mainly attributed to the hydrogen bonding and van der Waals interactions [36,37,38]. Firstly, the flavonoid structures contain several hydroxyl groups that can interact with XAD7HP through hydrogen bonding, which is a specific intra-molecular or inter-molecular interaction. Secondly, the van der Waals forces from the aqueous solvent system became the main driving force for polymeric XAD7HP in the sorption process. Therefore, the combination of these interactions contributes significantly to the adsorption and desorption processes, as indicated by the high adsorption/desorption capacities.

## 3. Materials and Methods

### 3.1. Chemicals and Reagents

Purified water was obtained from a Milli-Q system (Millipore Lab, Bedford, MA, USA). Sodium acetate, phosphoric acid, quercetin, sulfanilamide, and *N*-(1-naphthyl) ethylenediamine dihydrochloride were provided by Sigma (Aldrich, Germany). Aluminum chloride was purchased from HmbG Chemicals (Hamburg, Germany). Hydrochloric acid, dimethyl sulfoxide (DMSO), and sodium hydroxide were purchased from Merck (Darmstadt, Germany), whereas 95% ethanol was obtained from R&M Chemicals (Essex, United Kingdom). Sodium nitroprusside was purchased from Bendosen Laboratory Chemical (Bendosen, Norway).

### 3.2. Adsorbents and Pretreatment

Macroporous resins (Amberlite^®^ XAD4, Amberlite^®^ XAD7HP, and Supelite™ DAX-8) were purchased from Sigma (Aldrich, Germany). The physical properties of these resins are summarized in Table 5. To remove monomers and porogenic agents trapped inside the pores, the resins were soaked in 95% ethanol at a resin/solvent ratio of 1:20 for 24 h. The resins were then washed with deionized water and soaked again in 1 M sodium hydroxide for another 24 h after several washes with deionized water; then, the resins were soaked in 1 M hydrochloric acid for 24 h and washed again with deionized water. The resins were dried (60 °C) in a universal drying oven (model 100–800, Memmert, Schwabach, Germany) until constant weight was observed.

### 3.3. Preparation of OPL Extract

Mature OPLs were harvested from University Agricultural Park, Universiti Putra Malaysia (UPM). A voucher specimen (SK 3332/18) of the plant material was authenticated and deposited in the Herbarium of Institute of Bioscience, UPM. The leaflets were cut into small pieces of approximately 2.54 cm (an inch length) in size, and subjected to oven-drying at 35 °C until constant weight was observed. The dried OPLs were ground into fine powder, mixed with methanol/water (4:1), and vortexed at 3000 rpm for 30 s. The mixture was then sonicated for a further 30 min in an ultrasonic water bath (Branson 2510MT Ultrasonic Cleaner, Darmstadt, Germany) with a frequency of 40 Hz at 25 °C. The mixture was centrifuged at 4000 rpm for 15 min. After separation, the supernatant was concentrated using a rotary evaporator (Heidolph Instruments GmbH and Co.KG, Schwabach, Germany). The OPL extract was then freeze-dried using Labconco^®^ FreeZone Freeze Drier System (Kansas, MO, USA) for complete moisture removal.

### 3.4. Determination of Total Flavonoid Content

The total flavonoid content (TFC) of OPL extract samples (initial and after resin treatment) was determined using the aluminum chloride complex colorimetric assay as previously described by Formagio et al. [39], with some modifications. Test sample solutions (0.1 mg/mL) were prepared in methanol. For measurement, a 125-µL aliquot of the test sample solution was mixed with 375 µL of 95% ethanol, 25 µL of 10% aluminum chloride solution, 25 µL of 1 M sodium acetate solution, and 700 µL of distilled water in a 2-mL microcentrifuge tube. The reaction mixture was vortexed and incubated at 25 °C in an orbital shaker at a speed of 100 rpm for 40 min. The absorbance of the mixture was then recorded at 415 nm on a Tecan Infinite F200 Pro plate reader (Tecan Group Ltd., Männedorf, Switzerland). The analysis was carried out in triplicate, and the results were expressed in milligrams of quercetin equivalent per gram of extract (mg QCE/g extract).

### 3.5. Static Adsorption and Desorption Experiments

#### 3.5.1. Adsorption Kinetics of OPL Total Flavonoids on the Selected MARs

To investigate the adsorption and desorption kinetics of each of the selected MARs on OPL total flavonoids, a specific procedure was followed. The accurately weighed resin (0.1 g) mixed with 5 mL OPL extract solution was placed in a 15-mL centrifuge tube, tightly capped and carefully sealed, placed horizontally in a WiseCube WIG-10RL Precise Shaking Incubator (Wisd Laboratory Instruments, Wertheim, Germany), set to a shaking speed of 150 rpm. The adsorption process was conducted over 24 h at 25 °C. The experiment was carried out on OPL extract solutions of different pH (5, 7, and 9; adjusted with 1 M HCl or 1 M NaOH), in triplicate. At 0, 5, 10, 15, 20, 25, 30, 40, 50, 60, 120, 180, 240, 300, 360, 480, and 1440 min time points, 0.125-mL aliquots of the treated OPL extract were withdrawn for TFC determination. The adsorption kinetics equation for each resin was accordingly established for each.

#### 3.5.2. Adsorption Thermodynamics of OPL Total Flavonoids on the Selected MARs

To investigate the adsorption thermodynamics of each of the selected MARs on OPL total flavonoids, a specific procedure was followed. Five different 15-mL centrifuge tubes containing the accurately weighed resin (0.1 g) were firstly prepared. The resin in each tube was mixed with 5 mL of OPL extract solution (adjusted to pH 7) of different concentrations (5, 2.5, 1.25, 0.625, and 0.313 mg/mL, respectively), capped and sealed as before, and placed horizontally in a WiseCube WIG-10RL Precise Shaking Incubator, set to a shaking speed of 150 rpm. The adsorption process was conducted over 24 h, at different temperatures (25, 35, 45, and 55 °C) in triplicate. At the end of the experiment, aliquots of 0.125 mL of the reaction mixtures were withdrawn for TFC determination. The adsorption thermodynamics for each resin was accordingly established for each.

#### 3.5.3. Static Desorption of OPL Total Flavonoids from the Selected MARs

The adsorption experiment was repeated, separately for each resin, using the optimized conditions (pH, concentration, temperature, and time). As before, the experiments were conducted in 15-mL centrifuge tubes containing the accurately weighed resin (0.1 g). After reaching adsorption equilibrium, excess solutions were removed from each tube, followed by the addition of aqueous ethanol solutions of various concentrations (20%, 40%, 60%, 80%, and 95%) for the desorption process. Desorption was again performed in the WiseCube WIG-10RL Precise Shaking Incubator, set to a shaking speed of 150 rpm, at 25 °C, for 24 h. At the end of the experiment, aliquots of 0.125 mL of the reaction mixtures were withdrawn for TFC determination. The desorption capacity was calculated, and desorption ratio vs. solvent concentration plots were established for each resin, accordingly.

### 3.6. Dynamic Sorption Experiments

Dynamic adsorption and desorption experiments were carried out on a glass column (2.5 cm × 46 cm) wet-packed with 4.4 g (dry weight) of the selected resin (200 mL bed volume) at room temperature. A 5 mg/mL OPL extract solution was prepared by dissolving 750 mg of the extract in 150 mL of deionized water. The solution was filtered, adjusted to pH 7, and cautiously introduced and eluted through the column. Then, 10-mL eluates were collected and their TFC determined as before. The breakthrough and the saturation points are represented by 5% and 95%, respectively, of the ratio of the TFC of the eluting volume to the TFC of the original OPL extract (*C*/*C_o_*). The adsorbed column was then washed with 30 mL of deionized water and then eluted with aqueous ethanol at the optimized concentration, at a constant flow rate of 0.3 mL/min. Then, 10-mL eluates were collected and their TFC determined as before. The dynamic sorption experiments were all carried out in triplicate.

### 3.7. Equations Used in This Study

The adsorption/desorption capacities, kinetics, and thermodynamics model equations applied in this study were as follows:

(2) $$\mathrm{The}\;\mathrm{adsorption}\;\mathrm{capacity}:\;q_{e}=\frac{C_{o}-C_{e}}{W}\times V$$

(3) $$\mathrm{The}\;\mathrm{desorption}\;\mathrm{capacity}:\;q{\;}_{d}=\frac{C_{d}V_{d}\;}{W}$$

(4) $$\mathrm{The}\;\mathrm{desorption}\;\mathrm{ratio}:\;D=\frac{C_{d}V_{d}}{(C_{o}-C_{e})V}\times 100$$

(5) $$\mathrm{The}\;pseudo-\mathrm{first}-\mathrm{order}\;\mathrm{kinetics}\;\mathrm{model}:\;ln\left(q_{e}\;-\;q_{t}\;\right)=-kt+lnq_{e}$$

(6) $$\mathrm{The}\;pseudo-\mathrm{second}-\mathrm{order}\;\mathrm{kinetics}\;\mathrm{model}:\;\frac{t}{q_{t}}=\frac{1}{k_{2}{q_{e}}^{2}}+\frac{t}{q_{e}}$$

(7) $$\mathrm{The}\;\mathrm{particle}\;\mathrm{diffusion}\;\mathrm{kinetics}\;\mathrm{model}:\;q_{t}\;=\;k_{p}\;\cdot \;t^{\frac{1}{2}}+C$$

(8) $$\mathrm{The}\;\mathrm{Langmuir}\;\mathrm{equation}\;\mathrm{and}\;\mathrm{its}\;\mathrm{variable}\;\mathrm{form}:\;\frac{C_{e}}{q_{e}}=\frac{K_{L}}{q_{m}}+\frac{C_{e}}{q_{m}}$$

(9) $$\mathrm{The}\;\mathrm{Freundlich}\;\mathrm{equation}\;\mathrm{and}\;\mathrm{its}\;\mathrm{variable}\;\mathrm{form}:\;qe=K_{f}{C_{e}}^{\frac{1}{n}}$$

(10) $$\mathrm{The}\;\mathrm{Van}\text{’}s\;\mathrm{Hoff}\;\mathrm{equation}\;\mathrm{for}\;\mathrm{enthalpy}\;\mathrm{change}\;(\Delta H):\;lnK=-\frac{\Delta H}{RT}+A.$$

Here, *q_t_*, *q_e_*, and *q_m_* are, respectively, the adsorption capacity at time t, the equilibrium adsorption capacity, and the maximum capacity to form a monolayer, measured as mg/g dry resin. *C_o_*, *C_e_*, and *C_d_* are, respectively, the initial concentration, equilibrium concentration, and concentration in desorption solution (mg/mL). *V* is the volume (mL) of initial solution, *V_d_* is the volume of the desorption solution (mL), and *W* is the dry weight (g) of the resin used. *q_d_* is the desorption capacity, while *D* is the desorption ratio (%). *k*_1_, *k*_2,_ and *k_p_* are the respective rate constants of pseudo-first-order, pseudo-second-order, and particle diffusion kinetic models of adsorption. *C* is the constant for the particle diffusion kinetics model. *K_L_* is the affinity parameter between resins and flavonoids (mL/mg). *K_f_* is the adsorption capacity of the resins, and 1/*n* is the adsorption intensity of the resins. *R* is the universal gas constant (8.314 J∙mol^−1^∙K^−1^). *T* is the absolute temperature (K), and A is a constant. The Langmuir and Freundlich equations were used to describe the adsorption equilibrium and linearity fitting of each equation.

### 3.8. UHPLC–UV/PDA and UHPLC–MS/MS Analysis of Crude and Total Flavonoid-Enriched Extracts

Crude and total flavonoid-enriched OPL extracts were separated using a C_18_ reversed-phase Acquity UPLC^®^ BEH column (1.7 µm particle size and 2.1 mm i.d. × 100 mm length) from Waters (Wexford, Ireland) on a Thermo Scientific Ultimate 3000 with a PDA-3000 photodiode array detector and a thermostat column compartment which was maintained at 25 °C during UHPLC analysis. Gradient elution was performed with water/0.1% formic acid/0.063% ammonium formate (solvent A) and acetonitrile/0.1% formic acid (solvent B). The program gradient proceeded using the following sequence of solvent B percentages: 10% for 0–0.6 min, 10%–11% for 0.6–1.0 min, 11%–11.3% for 1.0–1.5 min, 11.3% for 1.5–5.5 min, 11.3%–11.4% for 5.5–8.0 min, 11.4%–11.8% for 8.0–8.2 min, 11.8%–12% for 8.2–12.0 min, 12%–10% for 12.0–13.0 min, and 10% for 13.0–18.0 min. The flow rate was constant at 0.40 mL/min. The UV detector was set to 340 nm. The extracts were prepared as 5 mg/mL and filtered through a 0.22-µm syringe filter (Sartorius AG, Goettingen, Germany) for UHPLC injection.

After going through the UV/PDA detector, the flow was split to allow only 200 µL/min of eluent into the electrospray ionization (ESI) source of MS. The MS analysis was done on a Q-Exactive Focus Orbitrap LC–MS/MS system. The eluent was monitored by ESI-MS under negative mode scanned from *m*/*z* 67.9 to 1000. ESI was conducted using a spray voltage of 4.2 kV. High-purity nitrogen gas was used as dry gas at a sheath gas flow rate of 40 (arbitrary units) and aux gas flow rate of 8 (arbitrary units). The capillary temperature was set at 320 °C, while the aux gas heater temperature was set at 0 °C. The peaks were assigned based on their molecular weights, fragmentation patterns, and maximum wavelengths with additional support from previous studies and standard online databases (freely available), such as the Human Metabolome Database (HMDB) at http://www.hmdb.ca/, and PubChem at https://pubchem.ncbi.nlm.nih.gov/. The peaks were compared between the crude and total flavonoid-enriched extracts based on peak intensity. The peak intensity was classified based on the area under the curve.

### 3.9. Determination of DPPH Free Radical Scavenging Activity

The 1,1-diphenyl-2-picrylhydrazyl (DPPH) free radical scavenging assay was performed as described earlier by Lee et al. [40], with some modifications. Test concentrations of the OPL extract ranging from 0.7 to 100 µg∙mL^−1^ were prepared by serial dilutions of a 0.1 mg/mL stock solution. A 50-µL aliquot of the test solution was mixed with 100 µL of DPPH (5.9 mg∙100 mL^−1^), mixed well and incubated in the dark. After 30 min, the absorbance was measured at 515 nm using a Tecan Infinite F200 Pro plate reader (Tecan Group Ltd., Männedorf, Switzerland). Scavenging activity (SA) was calculated according to the following equation:

SA % = ((A_o_ − A_s_)/A_o_) × 100% (11)

where A_o_ is the absorbance of the blank, and A_s_ is the absorbance of the test sample.

The assay was carried out in triplicate, and results were stated as IC_50_ value in µg∙mL^−1^. Quercetin was used as a positive control in the assay.

### 3.10. Determination of Nitric Oxide (NO) Free Radical Scavenging Activity

The nitric oxide (NO) free radical scavenging assay was conducted according to Abdul-Hamid et al. [41], with some modifications. Basically, 1 mg of OPL extract was weighed and transferred into 1 mL of dimethyl sulfoxide (DMSO), followed by dilution with DMSO to reach final concentrations ranging from 0.98 to 1000 µg∙mL^−1^. A 60-µL aliquot of the test solution was mixed with 60 µL of sodium nitroprusside (0.05236 g dissolved in 20 mL of 10 mM phosphate buffer saline), mixed well and incubated at 298 K for 150 min. After incubation, 60 µL of Griess reagent (a mixture of 0.1 g of sulfanilamide, 0.01 g of *N*-(1-naphthyl) ethylenediamine dihydrochloride, and 10 mL of 2.5% phosphoric acid) was added into the reaction mixture, and the absorbance was measured at 550 nm using a Tecan Infinite F200 Pro plate reader (Tecan Group Ltd., Männedorf, Switzerland). Quercetin was used as a positive control in the assay. Scavenging activity (SA) was calculated according to the equation 11.

The assay was carried out in triplicate, and results were stated as IC_50_ value in µg∙mL^−1^. Quercetin was used as a positive control in the assay.

### 3.11. Statistical Analysis

Minitab statistical software (Version 16, Minitab Inc, State College, PA, USA) and InStat V2.02 statistical package (GraphPad Software, San Diego, CA, USA) were used for all statistical analyses. The results were presented as means ± standard deviation with three replications. One-way analysis of variance (ANOVA) completed by Tukey’s test was applied to perform analyses to determine significant differences. The significant level was set to *p* < 0.05.

## 4. Conclusions

In this investigation, the first attempt of the evaluation of the sorption properties of three MARs for total flavonoids from oil palm (Elaeis guineensis Jacq.) mature leaf extracts was reported. The static adsorption data were analyzed with empirical equations, indicating that the kinetic data fit the pseudo-second-order kinetic model, while the isotherms could be properly explained by the Freundlich isotherm model. The adsorption of the respective resins was an exothermic and experienced physical adsorption process. Furthermore, XAD7HP and 80% aqueous ethanol were selected in the dynamic sorption studies as optimal adsorbent and desorbing solvent, respectively. After treating with XAD7HP packed in a glass column, the TFC enhanced from 136.69 to 487.97 mg QCE/g with a 3.57-fold increment. Moreover, UHPLC-UV/PDA and UHPLC-MS/MS analysis revealed that apigenin and luteolin derivatives were selectively adsorbed by XAD7HP. In addition, both the crude OPL extract and the flavonoid-enriched fraction possessed good DPPH and NO free radical scavenging activities. Strong hydrogen bonding and van der Waals forces between the flavonoids and cross-linked polymeric XAD7HP resin explained the findings. With this knowledge, a simple yet efficient method in the enrichment of total flavonoids from agricultural waste was developed. As a result, the enriched fraction of these total flavonoids can be formulated into healthcare products and foodstuff for improving human health, especially in combating diseases proven to be treatable with bioactive total flavonoids.

## Figures and Tables

**Table 1:** Pseudo-first-order and pseudo-second-order kinetics equations and related model parameters of OPL total flavonoids on the selected MARs.

Resins	pH	*q_e_* (exp)(mg/g)	Pseudo-First-Order			Pseudo-Second-Order			Intraparticle Diffusion		
			*k*_1_	*q_e_* (cal) (mg/g)	*R* ^2^	*k*_2_	*q_e_* (cal) (mg/g)	*R* ^2^	*C*	*k_p_*	*R* ^2^
XAD7HP	5	40.5667	0.0026	6.3726	0.5460	0.0120	38.0228	0.9999	31.7520	0.3707	0.6049
	7	48.1583	0.0034	10.1381	0.7732	0.0045	45.6621	0.9996	34.8670	0.5890	0.7467
	9	37.6979	0.0050	17.4023	0.9444	0.0016	36.2319	0.9956	17.2340	0.9540	0.8673
DAX-8	5	27.6583	0.0041	15.1727	0.7054	0.0014	25.7731	0.9969	6.4897	1.0203	0.7685
	7	37.3583	0.0027	19.7707	0.7933	0.0018	30.9598	0.9957	12.9690	0.9407	0.8157
	9	30.5958	0.0022	19.8004	0.7973	0.0016	22.9885	0.9886	6.9934	0.8093	0.8353
XAD4	5	13.1521	0.0034	11.3441	0.9153	0.0009	11.9474	0.9573	0.2973	0.4973	0.9422
	7	20.9875	0.0042	17.8553	0.8235	0.0008	18.3824	0.9184	2.2351	0.7137	0.9162
	9	10.4937	0.0052	8.9692	0.9312	0.0014	10.2459	0.9400	1.0913	0.4005	0.9677

**Table 2:** Langmuir model, Freundlich model, and thermodynamic parameters of OPL total flavonoids on the XAD4, XAD7HP, and DAX-8 resins.

Resins	*T* (K)	Langmuir Equation				Freundlich Equation			ΔH (kJ/mol)
		*q_m_*	*K_L_*	*R_L_*	*R_1_* ^2^	1/*n*	*K_f_*	*R_2_* ^2^	
XAD4	298	4.3497	0.4419	0.6706	0.9401	0.6894	3.6247	0.9351	−19.96
	308	5.0352	0.8489	0.5146	0.8882	0.7879	3.1212	0.9660	
	318	3.6088	0.7232	0.5544	0.8412	0.6507	2.1153	0.9802	
	328	3.0609	0.6670	0.5743	0.7856	0.6260	1.8461	0.9741	
XAD7HP	298	67.568	0.3108	0.7433	0.8218	0.7450	80.926	0.9967	−7.49
	308	61.350	0.3006	0.7496	0.8561	0.7381	74.068	0.9908	
	318	66.667	0.3600	0.7143	0.8821	0.7667	76.342	0.9943	
	328	86.207	0.6466	0.5819	0.9575	0.8429	74.790	0.9973	
DAX-8	298	39.370	0.3071	0.7456	0.8745	0.6328	37.051	0.9960	−13.41
	308	37.594	0.3195	0.7379	0.8269	0.5953	32.590	0.9845	
	318	30.960	0.2879	0.7576	0.9517	0.6238	29.689	0.9970	
	328	27.933	0.2961	0.7524	0.8951	0.6045	25.578	0.9912	

**Table 3:** Putative identification of compounds present in crude extract and enriched fraction using UHPLC–UV/PDA and UHPLC–MS/MS analysis.

Peak	*t_R_* (min)	*λ_max_*, (nm)	[M − H]^−^ (*m*/*z*)	Formula	Key MS/MS Fragments (*m*/*z*)	Class	Possible Compound	Peak Intensity	
								Crude	Fraction
1	0.65	270	341.0850	C_12_H_22_O_11_	161.0224, 119.0327, 113.0223, 101.0223, 89.0224	Sugar	Sucrose	+	−
2	0.71	268, 374	366.9911	C_14_H_8_O_18_	182.9911, 139.0014, 94.0276, 67.0170	Organic acid	Chelidonic acid dimer	+	−
3	0.83	258	191.0189	C_6_H_8_O_7_	111.0066, 87.0067, 57.0328	Organic acid	Citric acid	++	−
4	0.93	312	315.0697	C_13_H_16_O_9_	152.0092, 109.0195	Phenolic acid	Dihydroxylbenzoyl-*O*-hexoside	+	−
5	0.96	288, 310	299.0748	C_13_H_16_O_8_	137.0222, 93.0325	Phenolic acid	Hydroxylbenzoyl-*O*-hexoside	++	−
6	1.07	286, 324	331.0645	C_13_H_16_O_10_	168.0405, 153.0170, 125.0221	Phenolic acid	Galloyl-*O*-hexoside	++	−
7	1.84	286, 324	329.0528	C_14_H_18_O_9_	167.0326, 153.0092, 123.0440	Phenolic acid	Vanilloyl-*O*-hexoside	++	−
8	2.49	278	385.0744	C_17_H_22_O_10_	223.0603, 209.0279, 191.0174, 147.0272	Phenolic acid	Sinapoyl-*O*-hexoside	++	−
9	2.57	280, 310	289.0717	C_15_H_14_O_6_	205.0480, 151.0378, 137.0222, 125.0222, 109.0273	Catechin	Catechin isomer	+	−
10	3.17	272, 348	609.1411	C_27_H_30_O_16_	519.1104 489.0998, 429.0786, 399.0696, 369.0585	Flavone	Luteolin-6,8-di-*C*-hexose	++	++
11	4.26	280, 324	289.0717	C_15_H_14_O_6_	205.0480, 151.0378, 137.0223, 125.0222, 109.0274	Catechin	Catechin isomer	+	+
12	4.88	272, 336	593.1464	C_27_H_30_O_15_	503.1155, 473.1051, 383.0739, 353.0638	Flavone	Apigenin-6,8-di-*C*-hexose	++	+++
13	6.72	272, 346	609.1411	C_27_H_30_O_16_	489.1001, 429.0789, 399.0679, 369.0604	Flavone	Luteolin-6,8-di-*C*-hexose	++	+
14	7.10	272, 334	563.1359	C_26_H_28_O_14_	473.1053, 443.0949, 383.0742, 353.0639	Flavone	Apigenin-6-*C*-pentose-8-*C*-hexose	+	++
15	7.86	270, 348	447.0896	C_21_H_20_O_11_	357.0588, 339.0480, 327.0483, 297.0379, 285.0381	Flavone	Isoorientin (Luteolin-6-*C*-hexose)	+++	++++
16	8.52	272, 336	563.1359	C_26_H_28_O_14_	473.1051, 443.0949, 383.0741, 353.0638	Flavone	Apigenin-6-*C*-hexose-8-*C*-pentose	+++	++++
17	9.00	270, 350	447.0896	C_21_H_20_O_11_	357.0587, 339.0476, 327.0485, 297.0378, 285.0380	Flavone	Orientin (Luteolin-8-*C*-hexose)	+++	+++
18	9.87	270, 348	593.1464	C_27_H_30_O_15_	473.1049, 429.0792, 369.0590, 357.0589, 327.0485	Flavone	Luteolin-6-*C*-hexose- 8-*C*-deoxyhexose	++	+++
19	11.22	274, 334	563.1359	C_26_H_28_O_14_	503.1168, 473.1056, 443.0950, 383.0743, 353.0639	Flavone	Apigenin-6-*C*-pentose-8-*C*-hexose	+	++
20	11.60	272, 336	593.1464	C_27_H_30_O_15_	473.1067, 413.0846, 369.0590, 357.0589, 293.0434	Flavone	Luteolin-6-*C*-hexose- 8-*C*-deoxyhexose	++	++
21	12.44	270, 338	431.0947	C_21_H_20_O_10_	341.0639, 323.0529, 311.0536, 283.0589	Flavone	Vitexin (Apigenin-6-*C*-hexose)	++	++++
22	13.85	270, 338	431.0947	C_21_H_20_O_10_	341.0638, 323.0536, 311.0536, 283.0588	Flavone	Isovitexin (Apigenin-8-*C*-hexose)	++	++++
23	17.19	270, 338	577.1306	C_27_H_30_O_14_	457.1098, 413.0845, 353.0630, 341.0640, 311.0536, 293.0432	Flavone	Apigenin-6-*C*-hexose-8-*C*-deoxyhexose	++	+++

(−) = absence, (+) = low intensity, (++) = medium intensity, (+++) = high intensity, and (++++) = significantly high intensity.

**Table 4:** TFC and DPPH free radical scavenging activity (IC_50_) of OPL extract and enriched OPL fraction.

Sample	TFC (mg QCE/g Dried Extract)	Antioxidant Activity (IC_50_ µg/mL)	
		DPPH	NO
OPL extract	136.6896 ^a^ ± 0.3106	15.8767 ^a^ ± 2.8610	17.8367 ^a^ ± 3.3301
Enriched total flavonoid fraction	487.9729 ^b^ ± 0.9748	59.4767 ^b^ ± 4.4895	68.1300 ^b^ ± 7.1184

Values labelled with the different letters (a and b) are significantly (*p* < 0.05) different between OPL extract and enriched total flavonoid fraction.

**Table 5:** Physical properties of the selected macroporous resins.

Type	Surface Area (m^2^/g)	Particle Diameter (mm)	Average Pore Diameter (Å)	Matrix	Polarity
Amberlite^®^ XAD4	750	0.250–0.841	100	Styrene–divinylbenzene	Non-polar
Amberlite^®^ XAD7HP	380	0.250–0.841	300–400	Acrylic	Moderate polar
Supelite™ DAX-8	140	0.250–0.420	225	Acrylic ester	Moderate polar
